# Development and evaluation of social cognitive measures related to adolescent dietary behaviors

**DOI:** 10.1186/1479-5868-9-36

**Published:** 2012-04-02

**Authors:** Deborah L Dewar, David R Lubans, Ronald C Plotnikoff, Philip J Morgan

**Affiliations:** 1 Priority Research Centre in Physical Activity and Nutrition, School of Education, University of Newcastle, Callaghan Campus, Newcastle, Australia

**Keywords:** Social cognitive, Measures, Adolescents, Dietary behavior

## Abstract

**Background:**

This study aimed to develop and evaluate the reliability and factorial validity, of social-cognitive measures related to adolescent healthy eating behaviors.

**Methods:**

A questionnaire was developed based on constructs from Bandura’s Social Cognitive Theory and included the following scales: *self-efficacy*, *intentions* (proximal goals), *situation* (perceived environment), *social support*, *behavioral strategies*, *outcome expectations* and *expectancies*. The questionnaire was administered with a two week test-retest among secondary school students (n = 173, age = 13.72 ± 1.24). Confirmatory factor analysis was employed to examine model-fit for each scale using multiple indices including: chi-square index, comparative-fit index (CFI), goodness-of-fit index (GFI), and the root mean square error of approximation (RMSEA). Reliability properties were also examined (ICC and Cronbach’s alpha).

**Results:**

The reliability and factorial validity of each scale is supported: fit indices suggest each model to be an adequate-to-exact fit to the data; internal consistency was acceptable-to-good (α=0.65−0.79); rank order repeatability was strong (ICC = 0.81−0.89).

**Conclusions and implications:**

Results support the reliability and factorial validity of social cognitive scales relating to healthy eating behaviors among adolescents. As such, the developed scales have utility for identifying potential social cognitive correlates of adolescent dietary behavior, mediators of dietary behavior change and validity testing of theoretical models based on Social Cognitive Theory.

## Background

There is good evidence to indicate that many adolescents demonstrate poor dietary practices while failing to meet current dietary guidelines and recommendations [1-3]. Excessive consumption of energy-dense nutrient poor foods is a major contributor to weight gain during adolescence [2], highlighting the importance of programs to improve diet quality in this group. Unfortunately, interventions directed at this population have achieved limited success [4,5]. A poor understanding of the mechanisms of behavior change has been offered as an explanation for the lack of efficacy in dietary interventions targeting youth [6].

Evidence suggests that health behavior interventions guided by relevant theory (e.g. Social Cognitive Theory, Self-Determination Theory) are more effective in changing behavior than non-theoretical approaches [7,8]. These theories hypothesize that an intervention’s effects are achieved through underlying “mechanisms” or mediators (e.g. self-efficacy or perceived benefits) that operate in varying degrees to facilitate the pathway between an intervention and behavioral outcomes [9]. For example, the Social Cognitive Theory (SCT) [10] postulates that behavior change is influenced by a complex interaction, referred to as ‘reciprocal determinism’, that occurs between personal factors, environmental factors, and attributes of one’s behavior itself [9].

Testing the validity of theoretical models applied to behavioral interventions allows for the development and refinement of theory, which can support the design and delivery of more effective interventions. However, interventions targeting dietary behavior in children and adolescents rarely assess the theoretical mechanisms of dietary behavior change [6]. Furthermore, the limited research investigating theoretical mechanisms of dietary behavior change in youth has been compounded by the use of mediator measures with unknown, limited or poor psychometric properties [6]. Consequently, little is known about affective mediators of dietary behavior change in youth.

Interventions to improve dietary behavior in youth are often guided by SCT and there is support for social cognitive correlates of dietary behavior in adolescents [11-13]. To the authors’ knowledge no previous study has developed and tested a comprehensive range of social cognitive scales for *“healthy eating”* in adolescents. Establishing the psychometric properties of evidence-based healthy eating scales may contribute to an improved understanding of dietary behavior by providing a parsimonious framework for the evaluation of interventions. As such, the aim of this current study was to: (1) develop a questionnaire that assessed major constructs from Bandura’s SCT that relate to a variety of healthy eating behaviors based on current dietary guidelines [14] and (2) evaluate the reliability and factorial validity of these measures in an adolescent sample.

## Methods

### Development of scales and items

A series of qualitative and quantitative processes were used in the development of the scales. Initially, qualitative methods were employed to develop and refine the scales [15]. A review of the literature was carried out to examine the content and psychometric properties of existing measures of social cognitive constructs related to adolescent dietary behavior. Subsequently, a preliminary questionnaire comprising seven scales was developed where each scale was considered to represent a unidimensional construct derived from Bandura’s SCT [9]: *self-efficacy, intention* (i.e., proximal goals)*, situation* (i.e., perceptions of the physical environment), *behavioral strategies, social support* and *outcome expectations* (i.e., perceived benefits) and *expectancies* (i.e., value placed on benefits) relating to healthy eating. Intention is a key construct from the Theory of Reasoned Action [16], and in Bandura’s Social Cognitive Theory is considered to be a proximal goal [17].

An important objective was to develop measures that address behaviors, intentions and beliefs regarding *healthy eating*. A definition referent for *healthy eating*^a^ as guided by key current dietary guidelines and recommendations for adolescents in Australia [14], was provided for respondents in the questionnaire. Although it is acknowledged that other definitions for “healthy eating” could be used, the preventive behaviors included in the referent are based on the best available evidence for key nutrition behaviors that have been linked to ill-health [18-20].

A specialist panel comprising of four experts in the areas of nutrition, SCT and/or scale development were consulted to review and refine the preliminary scales. The four specialists were asked to (a) consider the content validity of each scale by examining how well assigned items contributed to the theoretical conceptualization of the construct being measured, (b) consider the suitability of response options according to the wording of respective items, (c) evaluate item comprehension, and (d) consider the potential for respondent burden.

Subsequently, a focus group was conducted in the Spring of 2009 with an adolescent sample (n = 10, age 14.1 ± 0.6 years) that matched the questionnaire’s intended audience. This was for the purpose of reviewing and refining the scales. Participants were consenting students from a non-government school in Grades 8 and 9. A semi-structured interview setting was adopted and digitally recorded where probing was used to examine respondents’ thought processes used in arriving at an answer and interpreting items, instructions sets and response options. Following the focus group and suggested changes made, the scales were returned to the expert panel for further review before the scales were administered to the study sample.

### Scales

#### Self-efficacy

For the nine-item self-efficacy scale respondents were asked to rate their confidence in personal ability to choose/eat healthy foods whenever a choice is provided using a six-point Likert-type scale (*1* = *strongly disagree; 6 = strongly agree), (e.g. I find it difficult to choose healthy meals or snacks when I am eating out with friends)*.

#### Intentions

Using a four-point Likert-type scale (*1 = Not at all true of me; 4 = Very true of me*), five items assessed intentions to adopt healthy eating behaviors. The common stem “*In the next three months do you …*” provided a time referent to direct respondents to regard their intentions for the short-term future *(e.g. …do you intend to eat healthier portion sizes during meals - for example, not eating till you feel full).*

#### Situation

Six items examined an individual’s mental representation of the food available in their home environment. Specifically, items examined the provision of healthy snacks, drinks and the availability of fruit and vegetables *(e.g. At home fruit is always available to eat – including fresh, canned or dried fruit).* A six-point Likert-type scale again examined the respondents’ level of agreement/disagreement with each item.

#### Social support

Seven items assessed the frequency of social support received from parents for healthy eating using a five-point Likert-type scale *(1 = never to 5 = always)*. A time referent was provided to encourage consideration of supportive behaviors received during the previous three months *(e.g. …how often did your parents prepare a healthy home-cooked dinner for you?)* Some items were modified from a previous social support for healthy eating scale [21].

#### Behavioral strategies

The behavioral strategies scale comprised 10 items which assessed the frequency *(1 = never to 5 = always)* at which various behavioral strategies were employed during the previous three months to reinforce healthy eating. Specifically, various methods for enhancing the enjoyment of healthy eating, setting goals for healthy eating, and self-monitoring eating behaviors were inquired about, *(e.g. … did you leave food on your plate once you felt full?).* One item was modified from an earlier *change strategies for healthy eating* measure [21].

#### Outcome expectations and expectancies

The five-item outcome expectations scale combined new items with modified items from established measures relating to dietary or physical activity behaviors [22,23]. The expectations scale assessed beliefs about the physical and cognitive benefits of healthy eating. The expectancies scale provided five corresponding personal evaluations of the importance of each expectations benefit*.* Respondents rated the expectation and expectancy statements on a six- (*1 = strongly disagree to 6 = strongly agree*) and four- *(1 = not at all important to 4 = very important)* point Likert-type scale, *(e.g. Healthy eating can help me to feel more energetic throughout the day; How important is feeling more energetic to you?”)*.

### Questionnaire administration

After approval was received from the University Research Ethics Committee, consent was obtained from the Principals of three non-government schools from the Newcastle/Central Coast region of New South Wales for their school’s involvement in the questionnaire’s administration. Consenting secondary school-aged students from predominantly middle-class backgrounds were recruited from these schools to complete a two week test-retest in the Autumn of 2010.

### Data analyses

Using SPSS 17.0, descriptive statistics were obtained for all variables including means (M), standard deviations (SD) and frequencies (f). The proportion of missing data was negligible (0.19%), hence mean substitution was the preferred imputation method employed rather than exclusion methods to manage incomplete data [24].

#### Reliability

SPSS 17.0 was used to conduct the reliability analyses. For each scale a one-way analysis of variance (ANOVA) was performed to determine differences between repeat administrations [Test 2 (T2) – Test 1 (T1]. To provide a coefficient of individual repeatability the 95% limits of agreement were calculated. Scores for the difference between test administrations (T2 – T1) were plotted against the test-retest mean [(T1 + T2)/2] for each individual, after which the range of differences falling within the mean of the differences ± 1.96 standard deviations was calculated [25,26]. Bivariate correlations between the test-retest difference and mean were also obtained. This ascertained if limits of agreement were consistent throughout the range of measurements, as indicated by a small and non-significant correlation.

Intraclass correlation coefficients (ICCs) provide a measure of rank order repeatability. For each scale, an ICC score ≥ 0.75 indicates excellent reliability [27]. Cronbach’s alpha coefficient was also calculated to estimate internal consistency for each scale, whereby acceptable values are >0.6 [28].

#### Factor analysis

Confirmatory factor analysis (CFA) was conducted in AMOS 17.0 to examine model fit for each of the scales. A non-significant chi-square result (*p* > 0.05) indicates a good fit of the model being examined. However, a rejection of the hypothesized model can be an indication that the chi-square it is too sensitive to sample size [29], implicating the need for additional model-fit indices to be examined. Hence the following model-fit indices were calculated from baseline (T1) data: chi-square index, the root mean error of approximation (RMSEA), goodness-of-fit index (GFI), adjusted goodness-of-fit index (AGFI) and the comparative fit index (CFI). The RMSEA is generally regarded a principal index in examining model fit [30], where scores ≤0.08, ≤0.06, and 0.0, signify acceptable, close, and exact fits, respectively [31]. To interpret GFI, AGFI and CFI indices, scores ≥ 0.9, ≥ 0.95 or equal to 1 denote adequate, good and exact fit of the model respectively [31]. If data showed multivariate non-normality (multivariate kurtosis value represented by a Mardia’s coefficient > 3) [32], the Bollen-Stine bootstrap procedure was employed to examine model fit where bias corrected regression coefficients are reported [33].

## Results

### Descriptive statistics

The study sample consisted of 173 secondary school students (age = 13.72 ± 1.24; 62% female), with backgrounds comprising Australian (80.3%), European (9.9%), Asian (3.5%), Middle Eastern (1.8%), African (1.1%) and other (3.4%). There were no statistically significant differences between genders for test-retest scores (T2 - T1) for any of the scales. Hence separate analyses by gender were not investigated. Table 1 presents results for scale means and standard deviations.

**Table 1:** **Questionnaire means, standard deviations (*SD*), item kurtosis values, and Mardia’s coefficient of multivariate kurtosis**

**Constructs**	**Description**	**Source of modified items**	**Range (No. items)**	**T1 (Baseline)**			**T2 (2 week retest)**		
				**Mean ± *(SD)***	**Item kurtosis**	**Mardia (*z*)**	**Mean ± *(SD)***	**Item kurtosis**	**Mardia (*z*)**
Self-efficacy	Participants were asked to rate confidence in their ability to adopt and overcome barriers to healthy eating behaviors. Scale: 1 = *Disagree a lot:* 6 = *Agree a lot.*E.g.: *“I find it easy to eat at least 3 servings of fruit each day”.*		1-6 (7)	4.07 ± (0.81)	−1.06 to 2.38	4.91*	4.25 ± (0.80)	−1.03 to 1.80	2.53
Intentions	Participants were asked to indicate their intentions to eat healthily, starting with the common stem “In the next 3 months do you intend to…” Scale: 1 = *Not at all true of me:* 4 = *Very true of me*. E.g.: *“…do you intend to choose low-fat foods and drinks whenever you have the choice?”*		1-4 (5)	3.11 ± (0.52)	−0.64 to 0.61	4.87*	3.20 ± (0.55)	−0.54 to 0.90	7.15*
Situation	Participants were asked to respond to statements about their mental representation of the physical environment influencing their ability to eat healthy foods. Scale: 1 = *Disagree a lot:* 6 = *Agree a lot.* Example item: *“At home there are healthy drinks available – e.g. cold water, sugar-free drinks, reduced fat milk”.*		1-6 (4)	5.42 ± (0.56)	−0.16 to 2.57	15.01*	5.43 ± (0.58)	0.03 to 3.83	20.97*
Behavioral strategies	Participants were asked to rate the frequency at which they rein- forced their own healthy eating behaviors through setting goals, self-monitoring and strategies for enhancing enjoyment, starting with the common stem “In the past 3 months how often…”. Scale: 1 = *Never*: 5 = *Always*. E.g.: *“…did you choose reduced-fat options when they were available?”*	[21]	1-5 (6)	3.24 ± (0.71)	−0.95 to 0.00	−0.02	3.33 ± (0.74)	−0.93 to −0.31	2.08
Social support	Participants were asked to rate the frequency with which family reinforced healthy eating through encouragement, role modeling, and accessibility to healthy foods, starting with the common stem “In the past 3 months how often…”. Scale: 1 = *Never*: 5 = *Always*. E.g.: *“…did your parents encourage you to eat fruit and/or vegetables?”*	[21]	1-5 (5)	4.29 ± (0.54)	−0.39 to 6.02	15.17*	4.30 ± (0.59)	−0.69 to 7.29	22.23*
Outcome expectations	Participants were asked to respond to statements about various benefits of healthy eating. Scale: 1 = *Disagree a lot:* 6 = *Agree a lot.* E.g.: *“Healthy eating (e.g. not skipping meals) can help to improve my concentration at school”.*	[22,23]	1-6 (5)	5.33 ± (0.51)	−0.06 to 4.02	19.05*	5.35 ± (0.49)	−0.67 to 6.54	16.15*
Outcome expectancies	Participants were asked to rate personal value placed on each corresponding outcome expectation item for healthy eating. Scale: 1 = *Not at all important:* 4 = *Extremely important* E.g.: *“How important is improving concentration at school to you?”*		1-4 (5)	3.40 ± (0.44)	−0.71 to 1.57	4.32*	3.43 ± (0.45)	−0.70 to 2.18	6.28*

*Note.* *Where Mardia’s coefficient for multivariate kurtosis indicate questionnaires which violate the assumption of multivariate normality (>3), the Bollen-Stine bootstrap procedure is employed to examine model fit.

### Confirmatory factor analysis

#### Self-efficacy

Preliminary analyses showed the original single-factor *self-efficacy* measure was a poor fit of the hypothesized model and that further refinement was necessary. An iterative process was employed to progressively remove items that were represented by unacceptable factor loadings and were found to contribute poorly to the model-fit indices. The final composite was reduced to seven items which resulted in an acceptable-to-good fit of the model (Table 2).

**Table 2:** Reliability results, model fit indices and factor loadings

	**Reliability results**				**Validity results**					
**Constructs**	***R***^***a***^	**95% LOM**	**ICC (95% CI)**	**Cronbach alpha**	**χ**^**2**^** (*p)***	**RMSEA**	**CFI**	**GFI**	**AGFI**	**Factor Loadings**
Self-efficacy	−0.03	−0.80 to 1.17	0.89 (0.85 to 0.92)	0.70	17.41 (0.04)	0.07	0.94	0.97	0.92	0.40 - 0.73
Intentions	0.08	−0.71 to 0.89	0.83 (0.77 to 0.87)	0.71	9.77 (0.08)	0.08	0.97	0.98	0.93	0.43 – 0.81
Situation	0.04	−0.88 to 0.91	0.81 (0.75 to 0.86)	0.79	0.90 (0.64)	0.00	1.00	1.00	0.99	0.44 – 0.86
Behavioral strategies	0.06	−0.84 to 1.00	0.88 (0.84 to 0.91)	0.75	6.69 (0.67)	0.00	1.00	0.99	0.97	0.43 – 0.75
Social support	0.15	−0.68 to 0.70	0.89 (0.85 to 0.92)	0.71	7.23 (0.20)	0.05	0.98	0.98	0.95	0.47 – 0.70
Outcome expectations	−0.06	−0.70 to 0.74	0.84 (0.79 to 0.88)	0.72	14.67 (0.01)	0.11	0.94	0.97	0.90	0.45 – 0.77
Outcome expectancies	0.03	−0.53 to 0.58	0.89 (0.87 to 0.92)	0.65	14.67 (0.54)	0.00	1.00	0.99	0.97	0.23 – 0.71

***NOTE:***^a^Bivariate correlations between the difference (T2-T1) and the mean [(T1 + T2)/2]; 95% limits of agreement calculated as the range of differences falling within the mean of the difference ± 1.96 SDs. ICC intra class correlation; CIs confidence intervals; χ^2^ = chi-square, *p* = probability; RMSEA root mean square error of approximation; GFI goodness of fit index; AGFI adjusted goodness of fit index; CFI comparative fit index.

#### Intentions

Analyses revealed the initial five-item *intentions* measure did not require further refinement. Table 2 shows the one-factor model demonstrated good model-fit as shown by adequate-to-good fit indices.

#### Situation

A reduced measure resulted in an improved four-item measure. The removal of two items produced fit indices that were a good or exact fit of the model (Table 2).

#### Social support

Model-fit results for the original seven-item measure did not satisfy all criteria. Two items were removed due their negative effect on factor loadings and fit indices. The final model resulted in fit indices which demonstrated an acceptable-to-good fit of the model.

#### Behavioral strategies

The original ten-item composite provided acceptable fit indices however, four items loaded poorly on the one-factor structure and so were removed to provide a more parsimonious measure. The reduction supported validation of the scale’s structure where fit indices demonstrated the measure was a good-to-exact fit of the model.

#### Outcome expectation and expectancy

Preliminary analyses indicated further refinement of the paired six-item *expectation* and *expectancy* measure was required. The removal of one pair of expectation/expectancy items which showed extreme platykurtic kurtosis resulted in considerable improvement in model fit for the expectancy measure. The final five-item expectations structure satisfied most model-fit criteria.

### Reliability analysis

Table 2 presents final reliability results. Bland-Altman analyses revealed favorable narrow limits of agreement for each scale. Non-significant bivariate correlations between the test-retest difference and test-retest mean indicated the limits of agreement were consistent throughout the range of measures for all scales. ICC scores for all scales indicated excellent rank order repeatability ranging from 0.81 (*situation)* to 0.89 (*self-efficacy, social support* and *outcome expectancy)*. Similarly, the internal consistency reliability of all measures proved adequate; Cronbach’s alpha values ranged from 0.65 *(outcome expectancy)* to 0.79 (*situation)*.

## Discussion

The aim of this study was to develop and evaluate the reliability and factorial validity of key social cognitive measures relating to adolescent dietary behaviors. Few studies have examined the validity of existing health behavior theories to explain and change dietary behavior in children and adolescents, and many studies that have examined potential mediators have used instruments with questionable psychometric properties [6]. The importance of using quality measures with strong psychometric properties for identifying hypothesized mechanisms of behavior change has been noted in the literature [34].

Overall, the results indicated each of the final scales presented to be a reliable measure showing acceptable factorial validity. All measures demonstrated at least acceptable internal consistency reliability (α > 0.60) [28] and excellent rank order repeatability (ICC > 0.75) [27], and factor analysis revealed the data to be an adequate fit of the hypothesized models. The final scales and their items are presented in Table 3.

**Table 3:** Scales and included items

***Self-efficacy scale*** ***CircleONEoption to indicate how much you agree or disagree with each statement. Whenever I have a choice of the food I eat…***																
						**Strongly Disagree**			**Disagree**	**Disagree Slightly**	**Agree Slightly**		**Agree**			**Strongly Agree**
1.	I find it difficult to choose low-fat foods (e.g. fruit or “lite” milk rather than “full cream” milk).					SD			D	DS	AS		A			SA
2.	I find it easy to choose a ***healthy*** snack when I eat in between meals (e.g. fruit or reduced-fat yoghurt).					SD			D	DS	AS		A			SA
3.	I believe I have the knowledge and ability to choose/prepare ***healthy*** snacks.					SD			D	DS	AS		A			SA
4.	I find it difficult to choose ***healthy*** meals/ snacks when I am eating out with my friends.					SD			D	DS	AS		A			SA
5.	I find it easy to eat at least 3 servings of fruit each day.					SD			D	DS	AS		A			SA
6.	I find it easy to eat at least 4 servings of vegetables/ salad each day.					SD			D	DS	AS		A			SA
7.	I find it easy to have healthy portion sizes during meals (e.g. not eating till I feel full).					SD			D	DS	AS		A			SA
***Intentions scale***																
**Please tick (✓) - In the next THREE MONTHS do you…**																
										**Not at all true of me**	**Not very true of me**		**Somewhat true of me**			**Very true of me**
1.	…**INTEND** to eat at least 3 servings of fruit each day?									□	□		□			□
2.	…**INTEND** to eat at least 4 servings of vegetables/ salad each day?									□	□		□			□
3.	…**INTEND** to choose low-fat foods and drinks whenever you have a choice?									□	□		□			□
4.	…**INTEND** to choose drinks and foods that are low in added sugar whenever you have a choice?									□	□		□			□
5.	…**INTEND** to eat healthier portion sizes during meals (e.g. not eating till you feel full)?									□	□		□			□
***Situation scale***																
***Circle ONE option to indicate how much you agree or disagree with each statement:***																
						**Strongly disagree**			**Disagree**	**Disagree slightly**	**Agree slightly**		**Agree**			**Strongly agree**
1.	At home there are healthy snacks available to eat.					SD			D	DS	AS		A			SA
2.	At home there are healthy drinks available (e.g. cold water in the fridge, sugar-free drinks, reduced-fat milk).					SD			D	DS	AS		A			SA
3.	At home fruit is always available to eat (including fresh, canned or dried fruit).					SD			D	DS	AS		A			SA
4.	At home vegetables are always available to eat (including fresh, frozen or canned vegetables).					SD			D	DS	AS		A			SA
***Behavioral strategies scale***																
***Circle ONE option for each question. In the past THREE MONTHS…***																
									**Never**	**Rarely**	**Sometimes**		**Often**			**Always**
1.	…did you choose reduced-fat options when they were available (e.g. “lite” milk, reduced-fat cheese and yoghurt)?								N	R	S		O			A
2.	…rather than choose sugary drinks such as fruit juice or soft drink, did you choose water or sugar-free drinks such as diet soft drink?								N	R	S		O			A
3.	…did you leave food on your plate once you felt full during a meal?								N	R	S		O			A
4.	…did you prepare healthy snacks and meals for yourself that were that were low in fat ***and*** low in added sugar?								N	R	S		O			A
5.	…did you try preparing new recipes for meals and snacks that were low in fat ***and*** low in added sugar?								N	R	S		O			A
6.	…did you do things to make eating fruits and vegetables more enjoyable (e.g. try a new recipe or blend fruit to make a fruit smoothie)?								N	R	S		O			A
***Social support scale***																
***Circle ONE option for each question. In the past THREE MONTHS how often…***																
									**Never**	**Rarely**	**Sometimes**		**Often**			**Always**
1.	…were fruit and vegetables available at home?								N	R	S		O			A
2.	…did your parents/caretaker make ***healthy*** snacks available (e.g. fruit or reduced-fat yoghurt)?								N	R	S		O			A
3.	…did your parents/caretaker prepare a ***healthy*** home-cooked dinner for you?								N	R	S		O			A
4.	…did your parents/caretaker encourage you to eat fruits and vegetables?								N	R	S		O			A
5.	…did you prepare ***healthy*** snacks or meals with your parents/caretaker?								N	R	S		O			A
***Outcome expectations and expectancies scale***																
**Please tick (✓) ONE option to indicate how much you agree or disagree with each benefit andhow important each benefit is to you:**																
1a. Healthy eating can reduce my risk for some illnesses and diseases (e.g. heart disease, diabetes, some cancers etc).																
□		□						□		□		□			□	
**Strongly Disagree**		**Disagree**						**Partly Disagree**		**Partly Agree**		**Agree**			**Strongly Agree**	
1b. How important is reducing your risk for illness and disease **to you**?																
□						□				□			□			
**Not at all important**						**Only slightly important**				**Important**			**Extremely Important**			
2a. Healthy eating can help me to feel better physically.																
□			□					□		□		□				□
**Strongly Disagree**			**Disagree**					**Partly Disagree**		**Partly Agree**		**Agree**				**Strongly Agree**
2b. How important is feeling better physically **to you**?																
□						□				□				□		
**Not at all important**						**Only slightly important**				**Important**				**Extremely Important**		
3a. Healthy eating can help me to control my weight.																
□		□						□		□		□			□	
**Strongly Disagree**		**Disagree**						**Partly Disagree**		**Partly Agree**		**Agree**			**Strongly Agree**	
3b. How important is controlling your weight **to you**?																
□							□			□				□		
**Not at all important**							**Only slightly important**			**Important**				**Extremely Important**		
4a. Healthy eating (e.g. not skipping meals) can help to improve my concentration at school.																
□		□						□		□		□			□	
**Strongly Disagree**		**Disagree**						**Partly Disagree**		**Partly Agree**		**Agree**			**Strongly Agree**	
4b. How important is improving your concentration at school **to you**?																
□					□					□				□		
**Not at all important**					**Only slightly important**					**Important**				**Extremely Important**		
5a. Healthy eating can help me to feel more energetic throughout the day																
□		□						□		□		□			□	
**Strongly Disagree**		**Disagree**						**Partly Disagree**		**Partly Agree**		**Agree**			**Strongly Agree**	
5b. How important is feeling more energetic **to you**?																
□				□						□				□		
**Not at all important**				**Only slightly important**						**Important**				**Extremely Important**		

Comparing the psychometric properties of the current scales with earlier measures of theoretical constructs of dietary behavior was challenging for a number of reasons. First, previous studies have focused on concurrent and criterion validity by comparing new scales to similar measures or actual dietary intake e.g. [21,35]. Alternatively, few studies have examined the factorial validity of dietary scales which is important for establishing the degree to which measures conform to their theoretical construct [36]. Second, tests of reliability were often limited to an assessment of internal consistency. Additional reliabilities such as rank order repeatability (i.e. ICC) and limits of agreement are rarely reported e.g. [35,37].

Finally the majority of existing measures have focused on a specific dietary behavior or intake e.g. [22,38]. For example, adolescent measures reported by Haerens et al., [39] included several social cognitive scales for social support, self efficacy and perceived benefits that were exclusively related to the consumption of a low-fat diet. These measures provide researchers with a suitable solution for assessment when interested in a specific dietary behavior or intake. However, they also have limited utility when more than one aspect of dietary behavior is of interest, in which case respondent burden may become a problem if the administration of several questionnaires is required. For this reason, the measures presented may provide a suitable solution for researchers interested in a more generalized set of dietary behaviors based on current dietary guidelines and recommendations for adolescents [14].

### Implications

Current findings have provided evidence for the reliability and factorial validity of seven scales designed to measure SCT constructs related to healthy eating in adolescents (*self-efficacy, intentions, situationbehavioral strategies, social support*, and *outcome expectations* and *expectancies*). Collectively these scales provide a parsimonious solution for researchers interested in understanding dietary behaviors based on current dietary guidelines and recommendations for this group [14]. As such, the scales presented have utility for identifying potential social cognitive correlates of healthy eating, mediators of dietary behavior change, and assessing the validity of theoretical models of dietary behavior change based on SCT.

Despite the strengths of this study, there are some limitations that should be noted. First, the sample was relatively homogenous. Further psychometric testing of these measures in more ethnically diverse populations may be warranted. Also, sample numbers were too small to conduct meaningful sub-group analyses for gender.

Second, the tests of validity used in the current study were not extensive. Future researchers are encouraged to test the concurrent and convergent validity of these scales by comparing them with similar validated measures and dietary behavior. For instance, there is potential to test each scale against percentage of energy intake from core and non-core foods. Core foods include breads and cereals, fruits and vegetables, dairy products and meats, while non-core foods are energy-dense nutrient poor foods such as fast foods and processed snack foods [40]. Core foods correspond with the questionnaire’s definition referent for “healthy eating” as per dietary guidelines [14] for children and adolescents.

Finally, future directions could employ additional factor analytical techniques such that: (1) a cross validation of the measurement models is examined by employing a multi-group analysis of factorial invariance (e.g. between different socio-economic and ethnic backgrounds), and, (2) a longitudinal analysis of factorial invariance of the measurement models is examined (i.e. across time). An assessment of multi-group and longitudinal invariance can determine if differences between groups or over time are the desired result of true differences in the latent construct being measured (e.g. due to an intervention’s effects), or are explained by problematic differences in the measurement properties of the questionnaire(s) due to a change in how respondent’s interpret items and their relations.

## Conclusions

The results of this study provide support for the reliability and factorial validity of social cognitive measures assessing: *self-efficacy*, *intentions* (proximal goals), *situation* (perceived environment), behavioral strategies, *social support*, *outcome expectations* and *expectancies* related to healthy eating behaviors in adolescents. As such, these scales are suitable for the identification of potential social cognitive correlates of adolescent dietary behavior, mediators of dietary behavior change and the testing of theoretical models based on the SCT.

## Endnote

^a^*Healthy eating: having at least****3****servings of****fruit****and****4****servings of****vegetables****each day; choosing foods/drinks which are****low in fat****(e.g. fruit, vegetables, reduced fat yoghurt and milk, lean cuts of meat, wholegrain breads); choosing foods/drinks that are****low in added sugar****(e.g. wholegrain breads, water, sugar-free (diet) drinks); carefully considering healthy portion sizes during meals (e.g. avoiding eating till you feel full during meal times).*

## Abbreviations

SCT, Social Cognitive Theory.

## Competing interests

The authors declare that they have no competing interests.

## Authors’ contributions

DLD conducted the focus group interview and piloting process for the scales. DLD and DRL performed the statistical analysis. DRL, PJM and RCP helped DLD to draft the manuscript. All authors were involved in the development of the scales, and read and approved the final manuscript.
